# Radiation Therapy for Advanced Mucinous Carcinoma of the Breast With a Malignant Wound: A Case Report

**DOI:** 10.7759/cureus.22017

**Published:** 2022-02-08

**Authors:** Yojiro Ishikawa, Rei Umezawa, Takaya Yamamoto, Noriyoshi Takahashi, Kazuya Takeda, Yu Suzuki, Keita Kishida, Kengo Ito, Maiko Kozumi, Kaneki Koyama, Keiichi Jingu

**Affiliations:** 1 Division of Radiology, Tohoku Medical and Pharmaceutical University, Sendai, JPN; 2 Department of Radiation Oncology, Tohoku University Graduate School of Medicine, Sendai, JPN

**Keywords:** refusal of standard treatment, malignant wounds, surgery refusal, mucinous carcinoma, breast cancer, radiation therapy

## Abstract

Patients with breast cancer who refuse standard treatment often suffer from malignant wounds due to the growth of local tumors. However, treatment strategies for patients with unresectable locally advanced breast cancer who refuse standard treatment remain unclear. Usually, such cases are treated with palliative irradiation and do not achieve local control by irradiation alone. This is the first case report discussing the role of high-dose local irradiation and the treatment course for a patient with a massive breast tumor (mucinous adenocarcinoma) who refused standard treatment. A 44-year-old female was diagnosed with mucinous carcinoma of the breast in the right breast (cT1N0M0, cStage I). She refused standard treatment for six years. She visited the emergency department because of acute bleeding from the right breast with malignant wounds. Macroscopically, the tumor in the right breast measured over 20 cm in diameter. The tumor was exudative, exhibited ulceration and slight bleeding, and emitted an odor. Imaging findings showed multiple lymph nodes and bone metastases, and the final diagnosis was stage IV breast cancer (cT4bN1M1). Although the surgeon recommended chemotherapy for breast cancer, the patient refused chemotherapy or other therapy due to concerns regarding treatment-related complications. Considering the symptoms of advanced breast cancer with malignant wounds, she finally agreed to receive radiation therapy (RT). We performed RT at 70 Gy in 35 fractions over seven weeks. The tumor-associated symptoms disappeared after RT. Three months after RT, the tumor had almost disappeared. We administered luteinizing hormone-releasing hormone agonists after RT. Two years after RT, she died due to multiple liver metastases and ascites; however, there was no disease progression in the right breast. High-dose RT for locally advanced mucinous carcinoma of the breast with malignant wounds is considered an effective therapeutic option.

## Introduction

Patients with breast cancer often have malignant wounds. Malignant wounds can be associated with strong odors, bleeding, exudates, or infections [1]. Systemic therapy, such as chemotherapy, is the first-line treatment for unresectable locally advanced breast cancer; however, some patients refuse standard treatment for breast cancer [2-5]. Patients who refuse standard treatment often suffer from malignant wounds due to the growth of local tumors which affects their quality of life (QoL).

Treatment strategies for patients with unresectable locally advanced breast cancer who refuse standard treatment remain unclear. Typically, such cases are treated with palliative irradiation; however, local control cannot be achieved by irradiation alone. This case is the first report discussing the role of high-dose local irradiation and the treatment course for a patient with a massive breast tumor (mucinous adenocarcinoma) who refused standard treatment.

This article was previously uploaded as a preprint on Research Square (https://www.researchsquare.com/article/rs-1024514/v1).

## Case presentation

A 44-year-old female presented with a gradually growing tumor in her right breast for the past six years. She was diagnosed with breast cancer in the right breast at a previous hospital (cT1N0M0, cStage I). A core needle biopsy revealed mucinous carcinoma (MC) of the hypercellular type. The specimen consisted almost entirely of MC, without any obvious solid papillary component. The tumor was estrogen receptor-positive, progesterone receptor-positive, and human epidermal growth factor receptor 2 (HER2)-negative; moreover, the Ki-67 index was 32%.

The patient had refused standard treatment for her breast cancer. She visited the emergency department of our hospital with complaints of severe pain and acute bleeding from the right breast six years after the initial diagnosis. She reported 1,500-2,000 mL of exudate from the tumor per day, which was associated with significant weight loss for a month. Due to excessive exudation, she needed to perform self-care on her right chest every five to six hours, which reduced her sleep time and limited her ability to go out. She had no medical or family history of breast cancer or ovarian cancer. There was no history of drinking or smoking.

**Figure 1:**
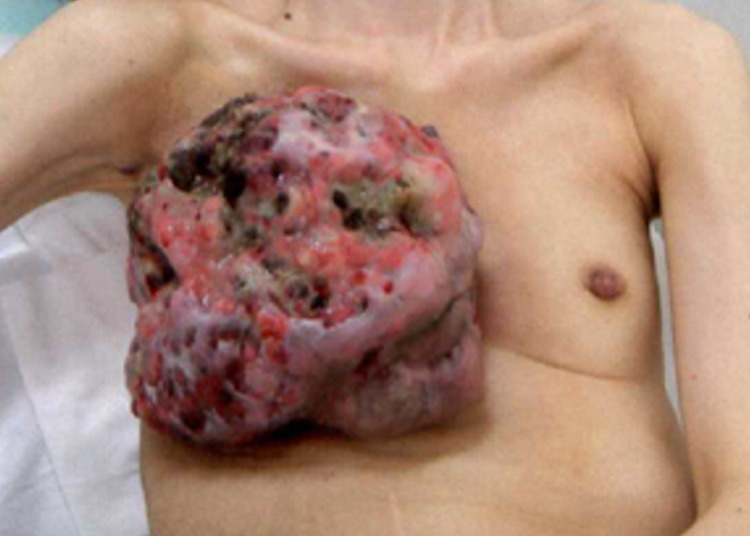
Macroscopic findings. Macroscopic findings revealed a tumor of over 20 cm in size on the right breast. The tumor was exudative, exhibited ulceration and bleeding, and gave off an odor. The patient had difficulty raising her right arm.

Macroscopically, the tumor in the right breast measured over 20 cm in diameter. The tumor was exudative, exhibited ulceration and slight bleeding, and gave off an odor (Figure 1).

**Figure 2:**
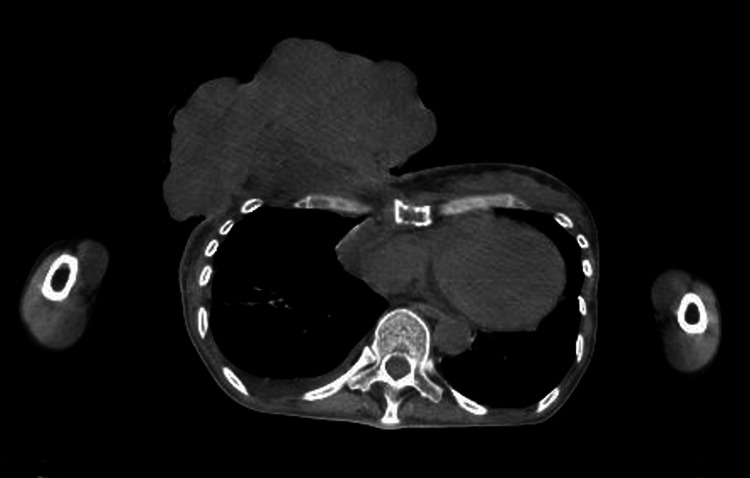
Axial enhanced computed tomography images. Images of the thoracic region show a tumor that had spread from the right chest wall to the outside of the skin. The lesion had not invaded the rib bone. Slight pleural effusion was observed in the right lung.

A computed tomography (CT) scan revealed a huge mass in the right breast (Figure 2).

**Figure 3:**
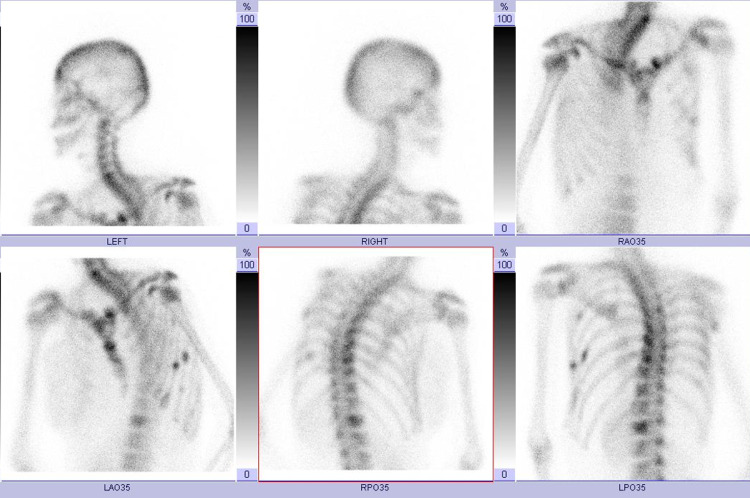
Bone scintigraphy images showing increased tracer uptake in some thoracic spines and ribs.

Although metastasis of multiple lymph nodes was observed, there was no metastasis in the liver and lungs on CT. Bone scintigraphy showed uptake in some thoracic spines and ribs (Figure 3). Her final diagnosis was breast cancer, stage IV (cT4bN1M1).

Although the surgeon recommended standard chemotherapy for breast cancer, the patient refused chemotherapy due to concerns about complications during chemotherapy. The patient also initially denied radiation therapy (RT). During the initial explanation of RT, the patient stopped presenting to the hospital. Although we recommended that the patient come to the hospital again, she refused RT for about a month and a half because of her work schedule.

Because refusal of treatment and refusal to come to hospitals had occurred at previous hospitals, we counseled the patient to discuss her thoughts on life before providing a technical explanation of RT. After patient counseling, we learned that the patient was looking for doctors who would recommend treatment without anticancer agents. However, she had repeatedly visited and interrupted the hospital course because none of the doctors offered the option of not using anticancer agents. The patient also said that she was unable to concentrate on her treatment because she was working in a special administrative department that did not have her own replacement.

We repeatedly listened to her thoughts about the treatment, and after careful dialogue regarding her anxiety about the treatment, we were able to obtain her consent to receive radiotherapy on the condition that anticancer drugs and endocrine therapy would not be administered. The patient was informed about palliative radiation therapy at another hospital before, and it was easier for her to accept the treatment because it did not require hospitalization and had fewer systemic side effects compared to other treatments. However, she did not necessarily want palliative treatment alone.

**Figure 4:**
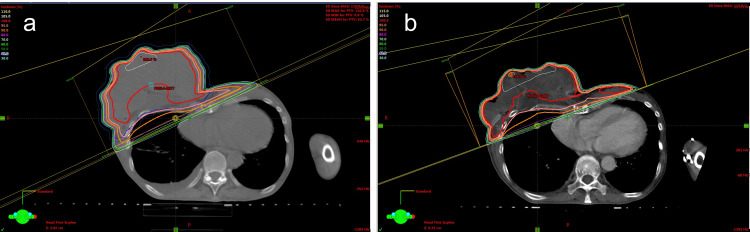
Dose distribution of radiation therapy. The primary tumor in the right breast was treated with 50 Gy (a) and boost radiotherapy with 20 Gy (b).

We performed RT at 70 Gy in 35 fractions over seven weeks. The patient received initial irradiation of 50 Gy in 25 fractions for the right breast without hormone therapy. The response of RT after 50 Gy was relatively good; however, symptoms such as pain, exudation, ulceration, and slight bleeding persisted. Therefore, we decided to add the boost. After the initial irradiation of 50 Gy, the patient received a sequential boost of 20 Gy in 10 fractions for breast cancer (Figure 4).

RT was delivered with 6-10 mV equipment via a multiple-leaf collimator by three-dimensional RT. Gross tumor volume (GTV) was defined as the primary tumor without lymph node and bone metastases based on pretreatment examination by CT. The clinical target volume (CTV) was defined as GTV plus 0.5 cm margins. The planning target volume (PTV) was CTV plus 1.0 cm margins. According to the cumulative dose-volume histograms, the ipsilateral lung volume receiving more than 20 Gy was 15%.

**Figure 5:**
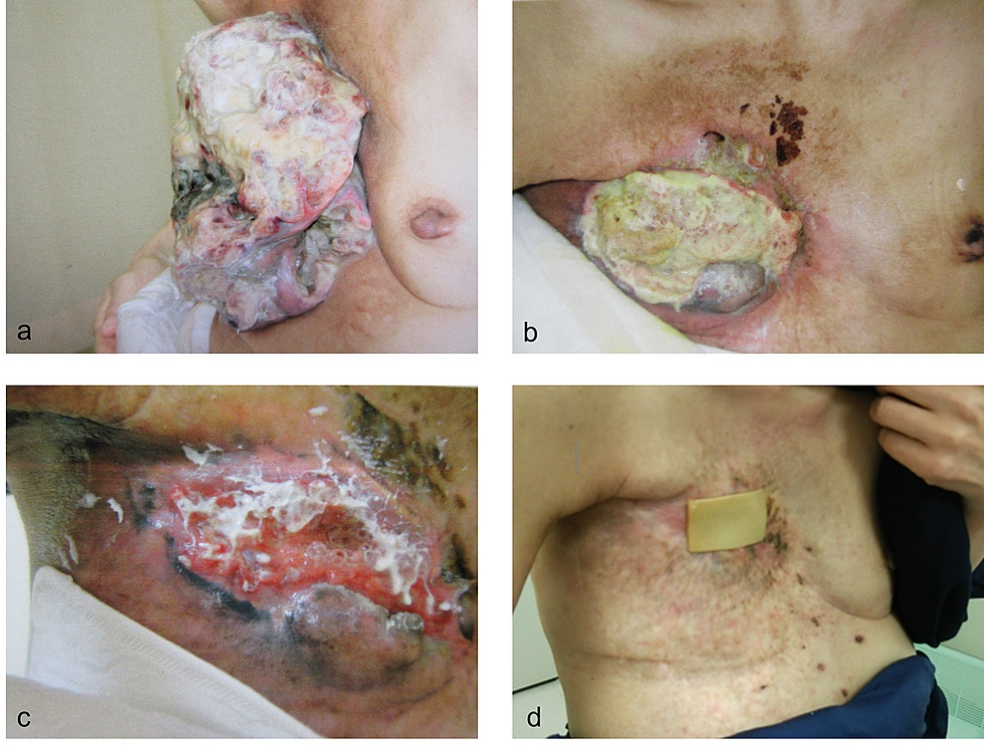
Macroscopic findings after RT. Macroscopic findings showed tumor regression one week after RT (a). The tumor had almost disappeared two months after RT, but necrotic lesion remained in the right breast (b). The wound gradually healed over three months (c). The wound was almost completely covered with skin four months after RT. Some pigmentation remained after RT (d). RT: radiation therapy

An acute side effect of grade 2 dermatitis according to the National Cancer Institute Common Terminology Criteria for Adverse Events version 4.0 occurred after RT; however, there was no acute or late complication of more than grade 3. The tumor-associated symptoms such as pain, exudation, ulceration, and bleeding disappeared after RT. One month after RT, the tumor regressed gradually and had almost disappeared three months after RT (Figure 5).

**Figure 6:**
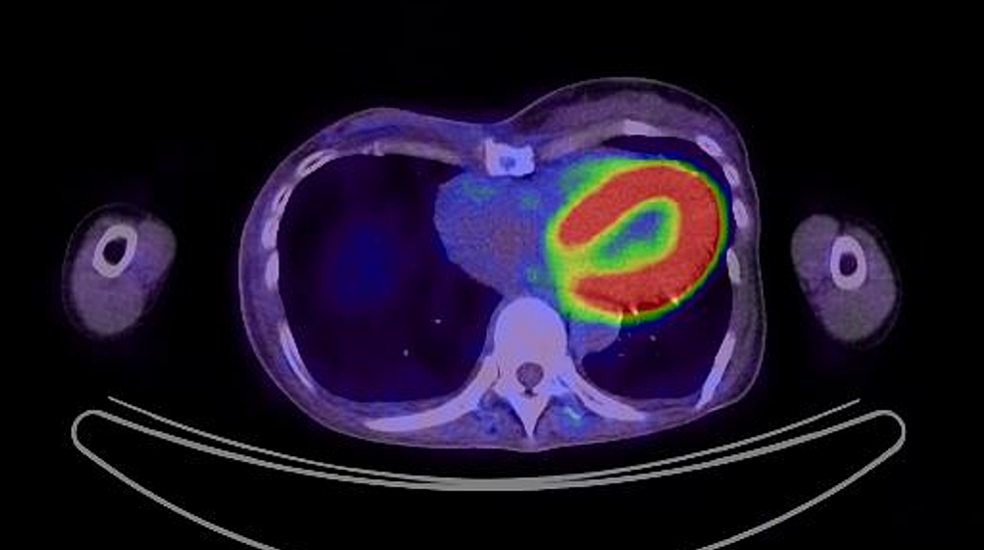
Positron emission tomography one year after radiation therapy. Radiation therapy resulted in the disappearance of fluorodeoxyglucose in the right breast.

Self-care of the right breast was no longer necessary, and her QoL had improved considerably. The patient did not agree to the anticancer drug treatment; however, she agreed to receive hormone therapy. We administered luteinizing hormone-releasing hormone agonists. Two years after RT, she died due to metastatic lesions in the liver and ascites; however, there were no symptoms or disease progression in the right breast (Figure 6).

## Discussion

Some patients with breast cancer refuse recommended follow-up tests or standard treatment. A retrospective study of 2,694 women with breast cancer showed that 7.2% of the patients refused provider follow-up advice [3]. According to a retrospective review of the Surveillance Epidemiology and End Results database between 2004 and 2013, the rate of surgery refusal was 0.64%. Moreover, age, ethnicity, marital status, disease stage, and lack of insurance were associated with a higher risk of refusal of surgery [2]. Although our patient did not refuse RT, Aizer et al. reported that 2,113 (0.9%) of 232,189 patients with malignancy refused RT despite recommendations by their physicians [4]. Therefore, it is notable that we were able to use RT for the patient despite her refusal of standard therapy. It is difficult to examine the reasons why the patient agreed to undergo radiotherapy this time. We believed that it was important for us to be close to the patient and address her anxiety about the disease and treatment.

Patients with advanced breast cancer often have malignant wounds. Malignant wounds are associated with a symptomatic burden [1]. Palliative therapy, such as chemotherapy or low-dose RT, is administered for unresectable locally advanced breast cancer with malignant wounds [6]. Additionally, Mohs paste or intratumoral hydrogen peroxide with RT has been used for patients with malignant wounds. Their use has become widespread for the primary goal of improving QoL [7,8]. However, the efficacy of Mohs paste or intratumoral hydrogen peroxide for breast cancer remains unclear.

The National Comprehensive Cancer Network guidelines and previous reports have recommended a single fraction of 8-10 Gy or 20 Gy in four to five fractions for patients with poor performance status (PS), and 30 Gy in 10 fractions or 50 Gy in 25 fractions for patients with good PS [6,9,10]. On the other hand, some studies have suggested that aggressive local treatment is associated with improved survival [11,12]. Recently, there have been several reports of hypofractionated radiotherapy with the following doses: 36.63 Gy/11 fractions, 30 Gy/5 fractions, 28.5 Gy/5 fractions, 27 Gy/5 fractions, 26 Gy/5 fractions, 40.05 Gy/15 fractions, and 42.56 Gy/16 fractions [13].

In this case, we achieved successful local control with a high-dose RT of 70 Gy for locally advanced breast cancer. It is not surprising that locally advanced breast cancer is controlled by surgery, chemotherapy, and RT; however, there have been few reports on successful treatment with RT without surgery or chemotherapy. Nakamura et al. reported the outcome of palliative RT for a breast cancer patient with skin invasion [10]. They did not recommend changing the radiation dose by pathological diagnosis. However, the pathology of the present case was probably associated with our outcome of locally advanced breast cancer. The pathology in our patient showed that the breast cancer was MC. The incidence of MC is 1-6% of all breast malignancies. The prognosis of MC has been reported to be better than other breast malignancies [14]. Pure MC is less likely than other types of invasive ductal carcinoma to spread to lymph nodes [15]. Our patient had fewer lymph node metastases despite the advanced stage. MC patients show high expression of hormone receptors and low expression of HER2. Similarly, our patient demonstrated high expression of hormone receptors and low expression of HER2. Adjuvant hormone therapy may have benefitted from the addition of hormonal therapy. In addition, the patient had a large tumor of more than 20 cm in diameter in her right breast; however, tumor size may not be a significant factor because mucin comprises most of the tumor volume [16].

Because this is a case study, it is difficult to define the indication for locally advanced MC of the breast with malignant wounds. Moreover, only the progress within two years could be observed in our patient. However, it is possible that patients with locally advanced MC of the breast with malignant wounds are treated only by palliative surgery, chemotherapy, or low-dose RT with hormone therapy, despite being potential candidates for high-dose RT. High-dose RT for locally advanced MC of the breast with malignant wounds is considered to be a therapeutic option in refusal of standard therapy.

## Conclusions

In this patient, we were able to control a huge mammary tumor by high-dose irradiation to a patient who had limited life expectancy due to advanced breast cancer. This is a case where low-dose irradiation was considered to alleviate bleeding, pain, and foul odor. However, in similar cases where the pathology is MC and local control can be judged to contribute to prolonged life expectancy, it can be an option for those seeking sustained relief of local symptoms.
